# Dissimilarity measures affected by richness differences yield biased delimitations of biogeographic realms

**DOI:** 10.1038/s41467-018-06291-1

**Published:** 2018-11-30

**Authors:** Adrián Castro-Insua, Carola Gómez-Rodríguez, Andrés Baselga

**Affiliations:** Departamento de Zoología, Genética y Antropología Física, Facultad de Biología, Universidad de Santiago de Compostela, Rúa Lope Gómez de Marzoa, 15782 Santiago de Compostela, Spain

## Introduction

Recently, Costello et al.^1^ (hereinafter COS) established 30 marine biogeographic realms, complementing similar work on terrestrial biotas^2^. However, in our opinion, their methods had two major limitations. First, the results were not reproducible based on the reported methods. Second, they defined regions using Jaccard similarity (*β*_jac_), but this index is not appropriate for the delimitation of biogeographic regions^3^ because it is affected by differences in species richness^4^. Therefore, sites with impoverished biotas are considered dissimilar and thus can be identified as a distinct biogeographic region, even if that region has no unique species. This bias is particularly problematic when the sampling effort is uneven, which COS acknowledge to be the case in their dataset^1^. Based on these limitations, we argue that the marine biogeographic realms published by COS^1^ should be reconsidered in light of the recommendations we provide here.

When defining biogeographic realms, the choice of the measure of dissimilarity between cells is fundamental. Indices that account only for the replacement component of assemblage dissimilarity^4–6^ and are thus independent of richness differences^7^, as Simpson’s dissimilarity index^8^ (*β*_sim_), must be selected^3^. COS^1^ also analyse their data using *β*_sim_, but argue that their results are robust to these alternative measures. However, we observe important discrepancies between their main result showing marine realms based on *β*_jac_ (see Fig. 2 in ref.^1^) and their map showing realms based on *β*_sim_ (see Fig. 3c in ref.^1^). For instance, in the former, the Atlantic Ocean is divided in two regions (northern and southern), and there is a separate region in the Indian Ocean, while in the latter all these regions seem to be lumped into one.

We used the dataset provided by COS^1^ in their Supplementary Material (species presence-absence in 5˚ × 5˚ cells) to test if we could define similar marine biogeographic regions by using *β*_sim_ between cells and well established procedures for delineating biogeographic regions^2,3^. We also used *β*_jac_ with the aim to reproduce the results of the authors. All analyses were conducted in R^9^ using the scripts provided in Supplementary Software 1. Given the large differences in sampling effort across cells, we removed cells with fewer than 5 species, following Costello’s et al.^1^ procedure (not explicit in the text, but it can be deduced from the cells missing in their maps (see Fig. 3c-d in ref.^1^). Nonetheless, alternative analyses based on the complete presence-absence table (Supplementary Fig. 1) yield regionalisations that are roughly similar to our main result. From the presence-absence table we obtained a matrix of dissimilarities between cells using function beta.pair() in package betapart^10^. We then performed a hierarchical cluster analysis on this matrix of dissimilarities, using function hclust() in R^9^. Unlike the selection of the dissimilarity measure, choosing the clustering algorithm is not straightforward, and there are two criteria that could be maximised^2^: (i) cluster internal coherence (minimising the dissimilarities within clusters and maximising the dissimilarities between them), and (ii) correlation between the original dissimilarities and the cophenetic distances in the dendrogram. The Ward clustering algorithm is intended to maximise the first criterion, and according to previous contributions^2^, average clustering performs well for the second criterion. We thus implemented both and assessed their performance as measured by ANOSIM tests^11^ (command anosim() in package vegan) for the first criterion, and as measured by the correlation (Spearman *ρ*) between *β*_sim_ dissimilarities and cophenetic distances for the second criterion. Ward clustering consistently performed better for the first criterion, yielding higher internal coherence of clusters than average clustering, for any number of clusters greater than 6. In turn, average clustering performed better than Ward clustering for the second criterion (Spearman *ρ* = 0.43 vs. *ρ* = 0.30, respectively). The average clustering method, as used by COS^1^ yielded unbalanced dendrograms, and as a result, most newly defined clusters consisted of only one cell or very few cells (see Supplementary Figs. 2–3). COS^1^ started with more than 200 clusters and then manually lumped them into 30 realms, an approach which implies that different realms are defined at varying levels of dissimilarity and introduces subjective decisions in the biogeographic classification. In contrast, Ward clustering yielded a more balanced regionalisation. In our view, the Ward algorithm is the most appropriate for this dataset, because internal coherence is the most relevant clustering criterion for regionalisation^2^, as the objective is to maximise the similarity within realms, and the differences between them. In contrast, preserving the distance between cells within realms is clearly less relevant for defining biogeographic regions.

**Fig. 1:**
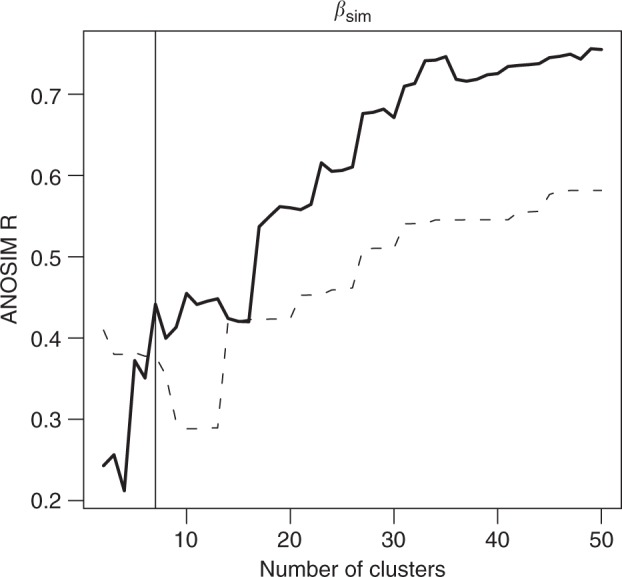
ANOSIM R values against the number of clusters in which the dendrogram is cut. The dendrogram was built using Simpson dissimilarity (*β*_sim_) between cells and Ward (solid line) or average (dashed line) clustering. The vertical grey line marks the number of clusters (*n* = 7) for which an increment does not yield relevant increments in ANOSIM R

Another critical step in biogeographic realm delineation is defining the number of clusters (realms). We assessed the significance of cutting the dendrogram resulting from the hierarchical cluster analysis into *n* clusters (*n* ranging from 2 to 50 clusters) by performing ANOSIM tests with command anosim() in package vegan^12^. In the *β*_sim_ dendrograms, we selected a value of *n* = 7 as the minimum value for which *n* + 1 did not cause a relevant increment in the ANOSIM R statistic (Fig. 1). We compared the maps produced with 7 defined clusters with those produced with 30 clusters as done by COS^1^.

**Fig. 2:**
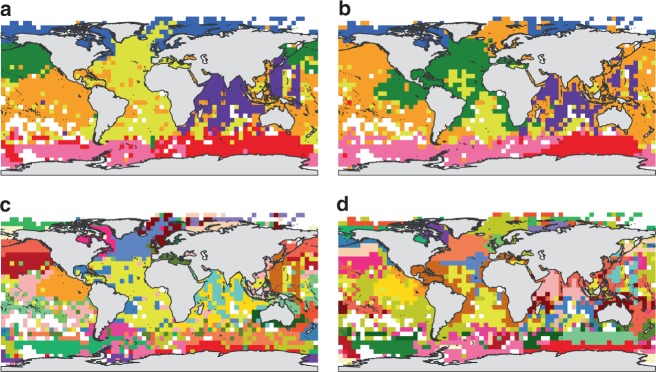
Regionalisation of marine assemblages in cells with 5 species or more. Maps represent marine realms yielded by Simpson (*β*_sim_: **a**, **b**) or Jaccard (*β*_jac_: **c**, **d**) dissimilarity indices and Ward clustering, defining 7 (**a**, **c**) or 30 (**b**, **d**) realms

When defining 7 regions, we found some important differences between using *β*_sim_ (Fig. 2a) or *β*_jac_ (Fig. 2c) regionalisations. The realms rendered by *β*_sim_ correspond roughly with the Atlantic, Arctic, and Indian Ocean, whereas the Pacific Ocean is split into North and South Pacific realms, and the Antarctic Ocean is divided into West and East Antarctic realms. However, when using *β*_jac_, a widespread region occupies the Pacific Ocean, and some parts of the Indian and Arctic Oceans, while the Atlantic Ocean is divided into a southern and northern regions (that also occupies part of the Pacidic Ocean). When defining 30 regions (Fig. 2b, d), the geographic coherence of realms is reduced, suggesting that the sampling noise is a relevant source of error at this level of similarity. We stress that there is no particular reason to define 30 regions (Fig. 1), but when doing so we find few similarities between the 30 realms defined by COS^1^ (see Fig. 2 in ref.^1^) and those yielded by the proper dissimilarity measure (*β*_sim_, Fig. 2a). Major differences are the distribution of realms in the Antarctic, North Atlantic, Pacific, and Indian Ocean.

In conclusion, we show that three major methodological decisions are critical for biogeographic regionalisation: dissimilarity index, clustering algorithm and number of clusters. Objective criteria are available to optimise the selection of clustering algorithms and number of clusters^2,3,13^ depending on the characteristics of the dataset. Regarding the selection of dissimilarity measures, a clear consensus has been recently reached about the need to use indices not affected by richness gradients^2,3,5,13,14^. If these methodological guidelines are not followed, biotic regionalisations will reflect richness gradients and sampling biases instead of the patterns we are aiming to capture (i.e., the replacement of species between realms with different biotas). The marine biogeographic realms proposed by COS^1^ suffer from these methodological problems and, therefore, do not provide a reliable regionalisation for use in conservation, biodiversity assessment, or climate change studies.

### Code availability

All analyses were conducted in R using the scripts provided in Supplementary Software 1 and 2.

## Electronic supplementary material

Supplementary Information

Description of Additional Supplementary Files

Supplementary Software 1

Supplementary Software 2

## Data Availability

The data are available in ref.^1^.
