# Supplementary material for: Dissimilarity measures affected by richness differences yield biased delimitations of biogeographic realms
Source: Nat Commun. 2018 Nov 30;9:5084. doi: 10.1038/s41467-018-06291-1 (PMC6269499; doi:10.1038/s41467-018-06291-1)
Supplement: Supplementary file 1 — Supplementary Information [file 41467_2018_6291_MOESM1_ESM.pdf]

Supplementary Material:

Correspondence: Dissimilarity measures affected by richness differences yield biased delimitations of biogeographic realms

Adrián Castro-Insua et al.

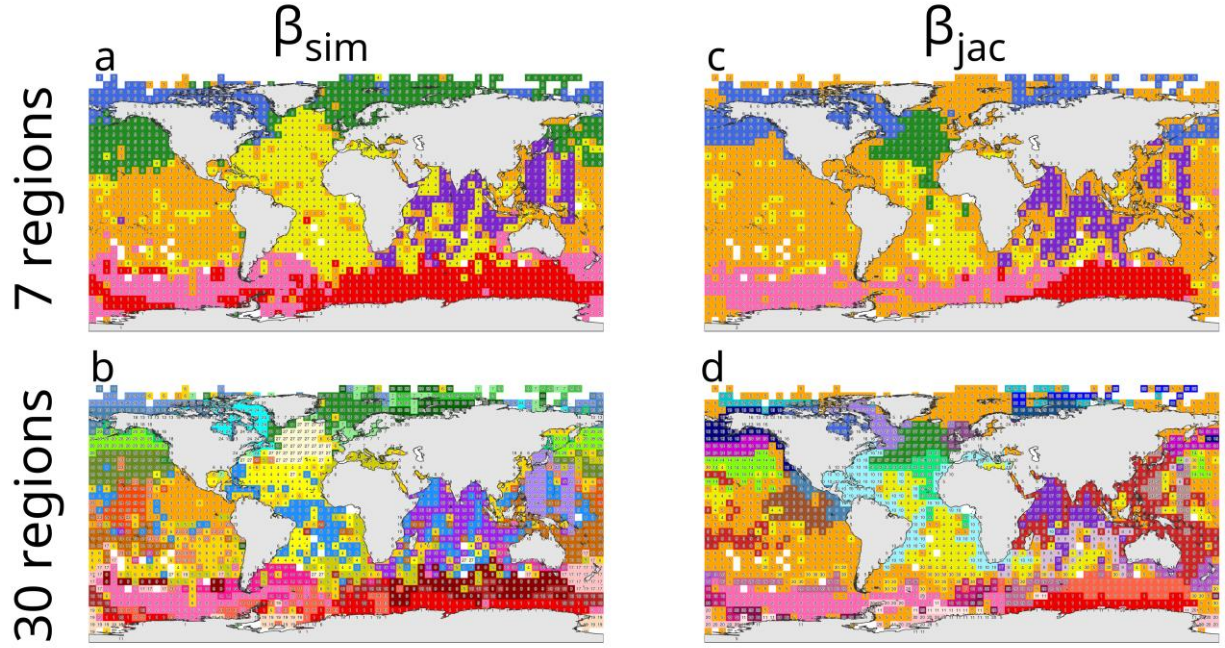

Supplementary Figure 1. Regionalisation of marine assemblages for the complete presence-absence table (i.e. without excluding cells with less than five species), using Simpson (β_{sim}: a, b) or Jaccard (β_{jac}: c, d) dissimilarity indices and Ward clustering, defining 7 (a, c) or 30 (b, d) realms.

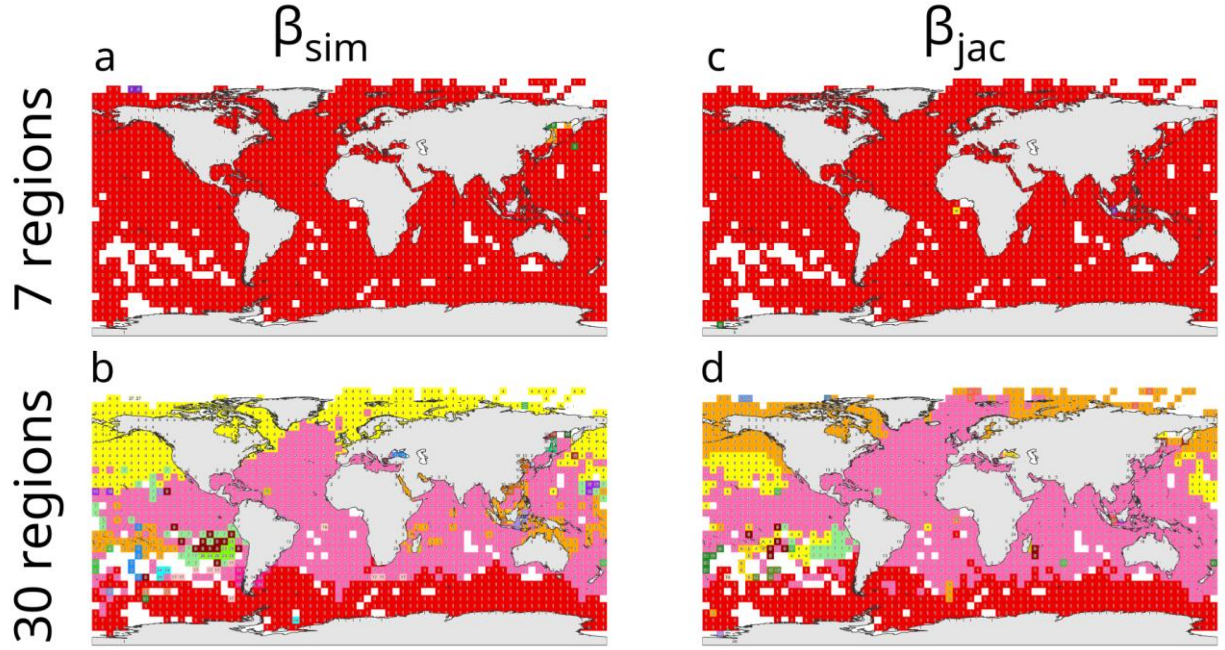

Supplementary Figure 2. Regionalisation of marine assemblages using Simpson (β_{sim}: a, b) or Jaccard (β_{jac}: c, d) dissimilarity indices and Ward clustering, defining 7 (a, c) or 30 (b, d) realms. Note that in many cases, regions consist of one or very few cells.

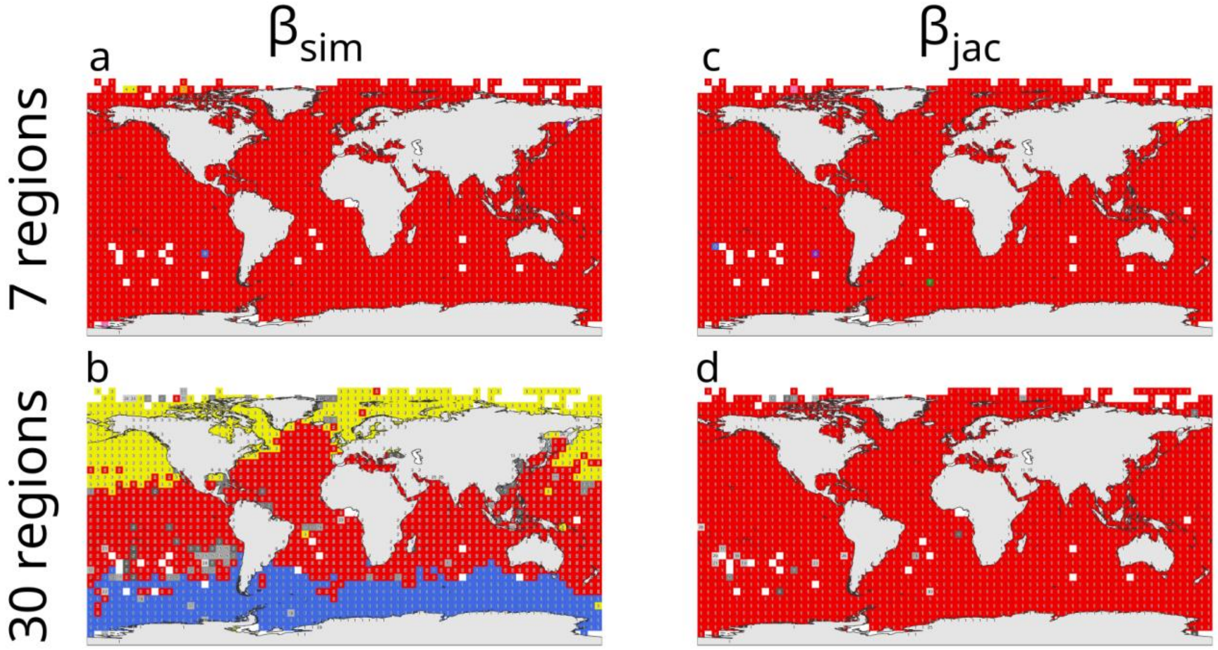

Supplementary Figure 3. Regionalisation of marine assemblages for the complete dataset (i.e. without excluding cells with less than five species), using Simpson (β_{sim}: a, b) or Jaccard (β_{jac}: c, d) dissimilarity indices and average clustering, defining 7 (a, c) or 30 (b, d) realms. Note that in many cases, regions consist of one or very few cells. When 30 realms are defined, realms comprising very few cells are coloured in grey tones.
