# Supplementary material for: Dissimilarity measures affected by richness differences yield biased delimitations of biogeographic realms
Source: Nat Commun. 2018 Nov 30;9:5084. doi: 10.1038/s41467-018-06291-1 (PMC6269499; doi:10.1038/s41467-018-06291-1)
Supplement: Supplementary file 2 — Description of Additional Supplementary Files [file 41467_2018_6291_MOESM2_ESM.pdf]

Description of Additional Supplementary Files:

Supplementary Software 1: code to conduct all the analyses and to produce all the maps reported in this paper.

Supplementary Software 2: R functions to translate between c-squares and lat/lon coordinates.
